# Case Report: Surgical management of a meningomyelocele in a cat

**DOI:** 10.3389/fvets.2026.1776519

**Published:** 2026-02-20

**Authors:** Kelly M. Muller, Zoe S. Daniels, Daniel M. Cimino, Jillian V. Hennessey

**Affiliations:** 1 The Schwarzman Animal Medical Center, New York, NY, United States; 2 College of Veterinary Medicine, The University of Arizona, Tucson, AZ, United States

**Keywords:** cat, congenital, meningomyelocele, myelopathy, neural tube defect, spina bifida

## Abstract

A 2. 5-year-old domestic shorthair cat was presented for evaluation of chronic progressive paraparesis and urinary incontinence characterized by leaking small amounts of urine and incomplete bladder emptying. General physical examination revealed a subcutaneous mass over the L4/L5 spinous process that was painful on palpation. Neurologic examination revealed non-ambulatory paraparesis with intact spinal reflexes, intact deep pain perception, and marked pelvic limb muscle atrophy. Magnetic resonance imaging showed a well-circumscribed tract extending from the epidermis through a split L5 spinous process to the level of the dorsal meninges with associated dorsal deviation of the spinal cord. These findings were consistent with spina bifida at L5 and an associated meningomyelocele. A dorsal laminectomy was performed for surgical decompression and the diagnosis of meningomyelocele was confirmed via histopathology. Postoperatively, the cat remained non-ambulatory and incontinent but had improved comfort. To the author's knowledge, this is the first report of surgical management of a meningomyelocele in a non-ambulatory cat.

## 1 Introduction

Neural tube defects (NTDs) encompass a collection of congenital malformations that are caused by failure of closure of the neural tube during embryogenesis (1). The neural tube is formed during neurulation when the neuroepithelium folds and fuses on midline. Folding is initiated at multiple sites along the craniocaudal axis, though the exact sites are not known in dogs or cats (2). Failure of closure of the rostral end of the tube leads to defects of the calvarium and cervical spine, and failure of closure of the caudal end causes defects in the thoracic, lumbar, and/or sacral spine (3).

In humans, NTDs are the second most common birth defect, affecting 1 in 1,000 American Caucasian births (3). NTDs are classified based upon the location and level of the defect. Open NTDs are characterized by exposure of neural tissue and leakage of cerebrospinal fluid (CSF) and closed NTDs retain coverage of the neural tissue (4).

The etiology of NTDs in both humans and animals is multifactorial; genetic factors, environmental factors, nutritional deficiencies, and exposure to toxins that affect mitosis have been implicated (3–13). There is significant evidence for genetic influence on NTDs. In humans, siblings have a 50 × higher risk for a NTD than the general population (4). Mutations in the *Vangl2* gene in mice and *NKX2-8* in Weimaraners and humans have been associated with NTDs (5, 6). Additionally, there is a high prevalence of NTD in Manx cats and Bulldogs, which further supports a genetic etiology (7, 8). There are multiple toxins reported to increase the risk of NTDs including prenatal exposure to heavy metals, pesticides, and some anti-fungal agents (9–11). In an experimental study, NTDs were induced in kittens when pregnant queens were given griseofulvin (12). Other environmental factors that have been associated with NTDs in humans include: maternal diabetes, maternal obesity, maternal use of anti-epileptic drugs, hyperthermia, hypervitaminosis A, and others (3). Folic acid supplementation has been shown to reduce the risk of NTDs and is commonly recommended to pregnant women (13).

Spina bifida is the most common manifestation of NTDs in humans and animals and is defined as the failure of fusion of one or more vertebral arches (14, 15). Protrusion of the meninges through a bony defect is termed a meningocele (MC). Protrusion of the meninges and nervous tissue through a bony defect is termed a meningomyelocele (MMC) (1). Reports of MMC are rare in veterinary medicine. In dogs, there are a few case reports, a short case series of bulldogs that were surgically managed, and a recent review paper describing MMC (8, 14, 16–20). There are two case reports of MMC in cats. In one case report, a MMC at the level of the coccygeal vertebrae was identified. The MMC was open and leaking CSF, leading to hypochloremia and hyponatremia. The cat underwent surgical correction and the electrolyte abnormalities resolved (21). In the second report, a MMC was identified at T13 and surgically managed in an ambulatory paraparetic cat. Post-operatively the cat had improved comfort and remained ambulatory (22).

Here, we present a case of a cat with chronic paraparesis, incontinence, and spinal pain that was diagnosed with a MMC on Magnetic Resonance Imaging (MRI) and treated with a dorsal laminectomy for decompression of the spinal cord. To the author's knowledge, this is the first report of surgical management of a meningomyelocele in a non-ambulatory cat.

## 2 Case description

A 2.5-year-old 7-kg neutered male domestic shorthair cat was presented for evaluation of chronic, progressive paraparesis and urinary incontinence. On initial presentation to an animal shelter at 9 months old, the cat was ambulatory paraparetic and had urinary incontinence characterized by leaking small amounts of urine and incomplete bladder emptying. Residual urine quantification performed at the shelter was elevated at 3 mL/kg. The cat was trialed on prazosin (0.14 mg/kg PO BID) and bethanechol (0.70 mg/kg PO BID) without significant change and was subsequently managed with manual bladder expression.

**Figure 1:**
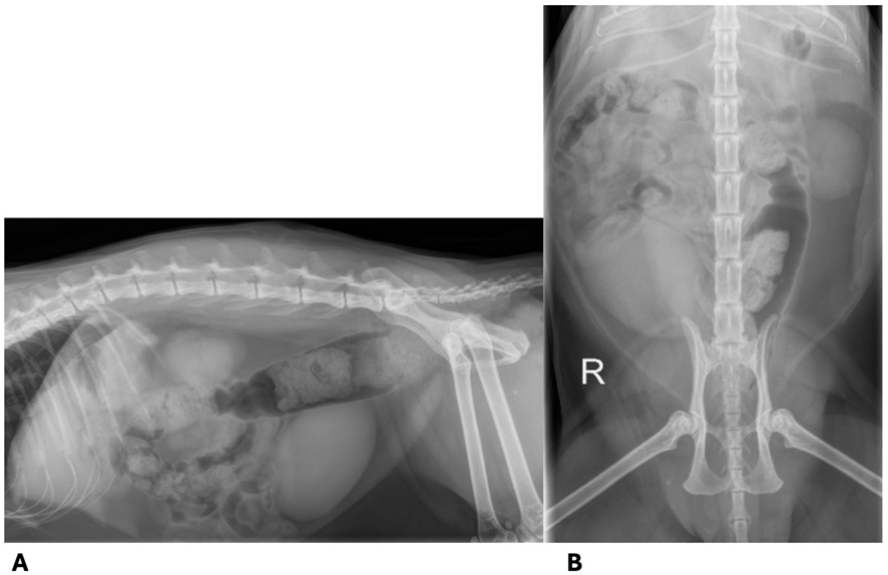
Spinal radiographs taken prior to presentation. **(A)** Lateral radiograph of the vertebral column. The lamina of L5 has an undulant cortical margin and there is a thin, soft tissue opaque linear tract extending dorsally from the L5 spinous process to the cutaneous margin. **(B)** Dorsoventral radiograph of the vertebral column. There is a well-defined, smoothly marginated ovoid lucency centered on the L5 spinous process.

Two months prior to presentation to the Schwarzman Animal Medical Center (AMC) neurology service, the cat became acutely painful and non-ambulatory with no known inciting cause. Diagnostics performed prior to presentation to the AMC neurology service included a complete blood count, serum biochemistry, urinalysis, toxoplasma titers, and spinal radiographs. Spinal radiographs showed an ovoid, well-defined, smoothly marginated, radiolucency centered on the L5 spinous process; an undulant cortical margin of the L5 lamina; a thin, soft tissue opaque linear tract that extends dorsally from the L5 spinous process to the cutaneous margin; and, mild colonic distension (Figure 1). These findings were suggestive of spina bifida. The remaining diagnostics were unremarkable. The cat was treated with a 3 week tapering course of prednisolone (starting at 0.40 mg/kg PO BID) and gabapentin (21 mg/kg PO TID) and there was no significant improvement in his gait, prompting referral.

**Figure 2:**
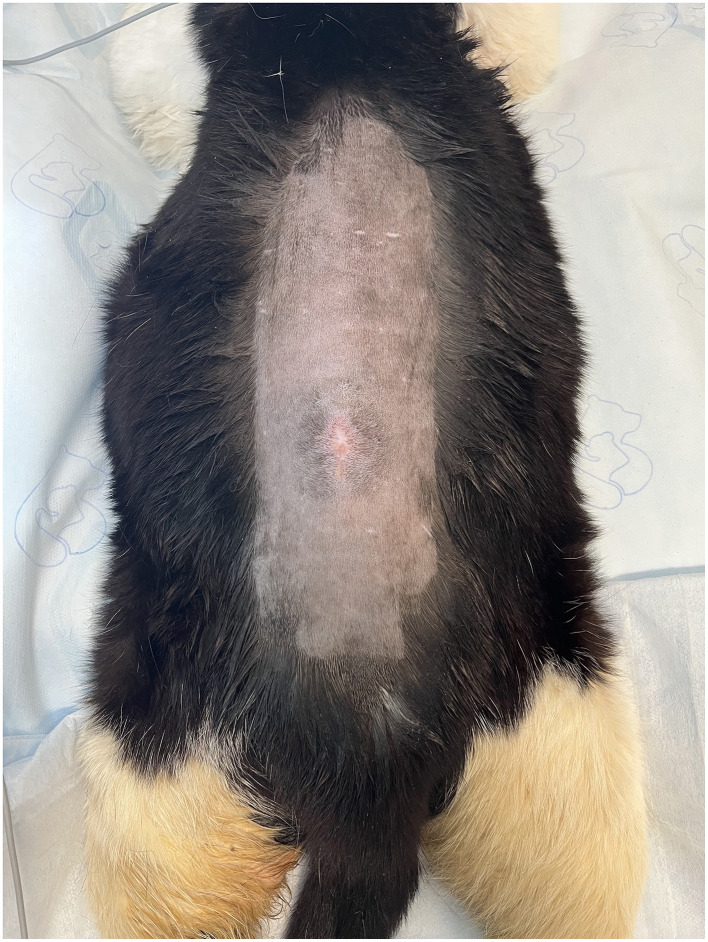
Pre-operative images displaying the shaved dorsum. Note the circular hair pattern surrounding the midline cutaneous mass.

General physical examination upon presentation to AMC revealed a small, soft, bladder, and a subcutaneous 1 cm × 1 cm mass over the L4/L5 dorsal spinous process. The cat was reactive and apparently painful on palpation of the mass (Figure 2). The remainder of the general physical examination was unremarkable. On neurologic examination, the cat was non-ambulatory paraparetic with moderate motor function in both pelvic limbs; absent conscious proprioception in both pelvic limbs; intact patellar, withdrawal, and perineal reflexes; intact deep pain perception; and moderate atrophy of the pelvic limb musculature. The cat's mentation, cranial nerve examination, and thoracic limbs appeared normal. The neurologic findings were most consistent with a T3-L3 myelopathy. The urinary incontinence may be explained by a concurrent L4-S3 myelopathy or detrusor muscle injury secondary to overdistension.

Repeat complete blood count and serum biochemistry revealed no remarkable abnormalities. Fine-needle aspirate of the subcutaneous mass over L4-L5 was consistent with adipose tissue.

**Figure 3:**
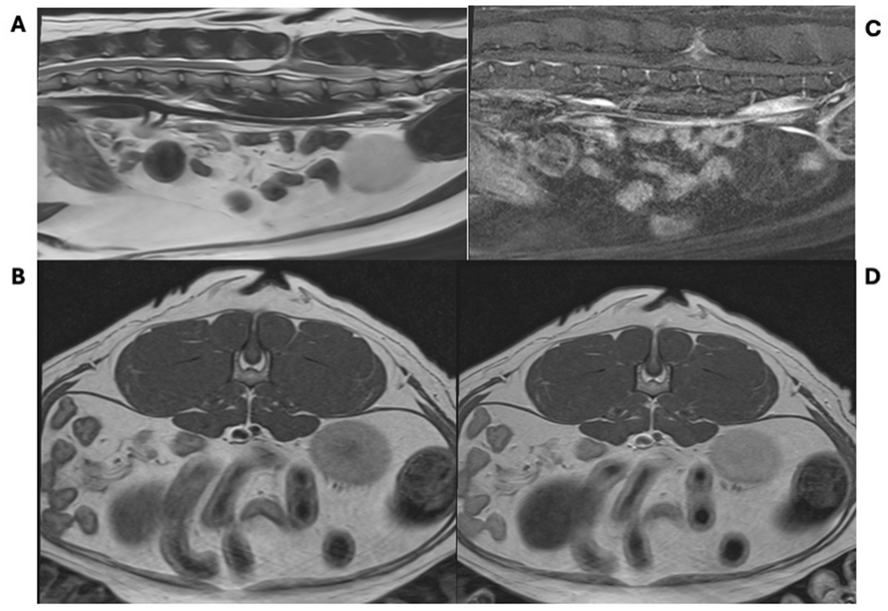
Magnetic resonance images of a cat with a meningomyelocoele. Sagittal T2-weighted **(A)**, transverse T1-weighted pre-contrast **(B)**, sagittal T1-weighted post-contrast **(C)**, and axial T1-weighted post-contrast **(D)** images showing a contrast enhancing tract through the L5 dorsal spinous process with marked syringohydromyelia at the level of L4, mild intramedullary contrast enhancement at L5 consistent with myelitis, and gas distension of the colon.

The cat was then anesthetized for an MRI of the thoracolumbar spine (inclusive of the first thoracic through first caudal vertebrae). The study was performed with a 1.5-T scanner (Magnetom Sola; Siemens Healthineers, Malvern, PA, United States) and the following sequences were obtained: T2-weighted (T2W) images in the sagittal and transverse planes; T2-DIXON images in the dorsal plane; and T1-weighted (T1W) DIXON images before and after the injection of a gadolinium-based contrast agent (Omniscan; GE Healthcare, Chicago, IL, United States) in sagittal, dorsal, and transverse planes. MRI revealed a well-circumscribed tract that extended from the epidermis ventrally through a split L5 spinous process to the level of the dorsal meninges (Figure 3). The tract was hypoattenuating on T1W and T2W and peripherally contrast-enhancing. The associated spinal cord was projected dorsally into the defect. Additionally, there was marked syringohydromyelia at the level of L4, mild intramedullary contrast enhancement at L5 consistent with myelitis, and gas distension of the colon. The remainder of the imaged spinal cord and abdomen were unremarkable. These imaging findings are consistent with spina bifida at L5 with an associated meningomyelocele and tethered cord.

**Figure 4:**
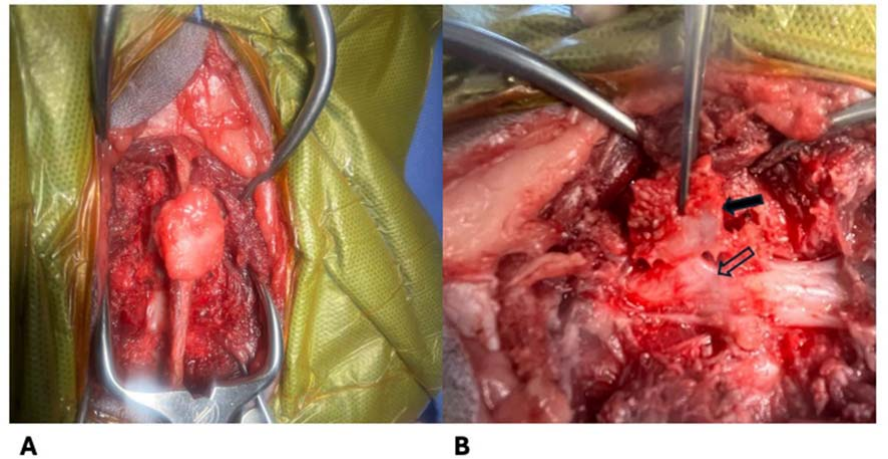
Intraoperative images of the meningomyelocoele. **(A)** Dorsal view of the mass attached to the dorsal spinous process **(B)** Lateral view of the mass (solid arrow) as it connects to the spinal cord (outlined arrow) prior to removal.

The cat was taken to surgery 2 weeks later and an L4-L5 dorsal laminectomy and durotomy were performed with removal of the L5 meningomyelocele from the level of the epidermis to the dura (Figure 4). An ellipsoid incision was made in the skin using care to avoid the region of suspected cutaneous and subcutaneous involvement. A stay suture was placed in the subcutaneous portion of the meningomyelocele tract and a combination of sharp and blunt dissection was utilized to separate the meningomyelocele from the surrounding tissue to the level of the dura. The attachment of the meningomyelocele to the dura was sharply transected. A durotomy was performed from the level of the meningomyelocele to the cranial aspect of L4 and a significant volume of CSF was released at high velocity. Adhesions of the pia to the dura were broken down using an Apfelbaum round dissector. The dural defect was covered with Biosis dural replacement and gelfoam sequentially.

Histopathology of the removed tissue showed a foci of well-differentiated fibrocartilaginous connective tissue with embedded peripheral nerves and vascular structures in close association with bone and skeletal muscle. Culture of the tissue was negative. These findings are diagnostic for a myelomeningocele.

Following surgery, the cat received a second tapering course of prednisolone (starting at 0.40 mg/kg PO BID) that was discontinued 3 months after surgery and remained on gabapentin (21 mg/kg PO TID). Six-months after surgery, the cat remained non-ambulatory paraparetic with intact deep pain perception and urinary incontinence, but he appeared comfortable on examination, more willing to move using his thoracic limbs, and was no longer reactive to palpation of the dorsal lumbar spine in the region of the excised mass.

## 3 Discussion

Meningomyelocele (MMC) is a rare NTD characterized by protrusion of the meninges and nervous tissue through a bony defect. Animals affected by a MMC are typically presented at a young age with clinical signs related to the location of the disease (1, 14). The lumbar spinal cord is most commonly affected, and associated clinical signs include a visible mass or dimple on dorsal midline, paraparesis, ataxia, fecal and urinary incontinence, and hyperesthesia (1, 7, 8, 16–20). If the MMC is open to the environment, there is risk of infection and bloodwork changes can include hypochloremia and hyponatremia due to continual loss of cerebrospinal fluid (1, 21). The cat described in the current report had a meningomyelocoele at the L5 spinal segment and marked syringohydromyelia at L4, but had a T3-L3 neurolocalization based on intact pelvic limb and perineal spinal reflexes. This discrepancy in localization may be due to concurrent pathology more rostral in the spinal cord that was not appreciated on the MRI, or there may be enough contribution of the L6 nerve root to the femoral nerve that a decreased patellar reflex was not observed (23).

Diagnosis of MMC relies upon advanced imaging. Radiographs can identify vertebral malformations consistent with spina bifida and myelography may show widening of the subarachnoid space; however, MRI is the ideal imaging modality to determine which neural tissues are involved in the defect and to identify secondary spinal cord changes (1). The radiographic changes in this case were subtle and were not appreciated on initial review. A definitive diagnosis of spina bifida and involvement of neural tissue was based on MRI, and the final diagnosis of MMC required histopathology.

There are reports of both medical and surgical management of MMC in veterinary and human literature (16–22). Medical management for MMC in animals includes analgesia, cleaning of cutaneous lesions, and antibiotics as needed for concurrent infection most commonly associated with an open NTD (1, 7, 21). The risks of medical management include neurologic worsening and infection (1, 7, 21). Various surgical techniques have been reported in animals, the most common of which include dorsal decompression, durotomy to remove the abnormal tissue, and dissection to release tethered tissues (1, 16–22). Reported surgical outcomes are variable with most animals exhibiting no change in ambulation or continence and a small subset exhibiting improvement or complete resolution of signs (16–22). The goals of surgical intervention should therefore be to prevent further neurologic decline, reduce the risk of infection, and improve comfort (1). The cat described here had improved comfort, characterized by resolved reactivity on spinal palpation, following surgery, which is likely related to resolution of tethering of the spinal cord.

In humans with MMC, early surgical intervention is recommended. There is evidence that closure after 72 h of birth is associated with increased complications and closure prior to 26 weeks of gestation is associated with improved neurologic outcomes (24). Both *in-utero* and postnatal surgical repair have been reported, with most surgical techniques including removal of the protruding tissue, detethering of underlying neural tissue, and occasional placement of ventriculoperitoneal shunt, cyst-subarachnoid shunt, or other shunt depending on the location of the defect (25–28). Medical management for MMC may be recommended to people who are more mildly affected or diagnosed at a later age and most commonly includes a combination of physical therapy and orthotics (29). The benefit of early surgical intervention highlight the need for early screening for congenital abnormalities in both humans and animals.

An animal with a T3-L3 myelopathy would be expected to have an upper motor neuron bladder. However, the incontinence described in this case as well as the clinical finding of a small, soft bladder is most consistent with a lower motor neuron bladder (30, 31). Given the lack of other lower motor neuron signs and the increased residual urine volume, the incontinence in this case is suspected to be due to detrusor muscle injury, likely due to chronic, previous overextension. Urinary incontinence has a significant negative impact on the quality of life of both an owner and pet, and it increases the risk of urinary tract infections and urine scalding (30, 31). In a case series of surgical management of MMC in bulldogs, only 2/6 dogs had improved urinary and fecal continence following surgery (8). There is only one report in veterinary medicine of complete resolution of fecal and urinary incontinence following surgical management of MMC in a 7-week-old Yorkshire terrier (20). In the current study, there was no change in continence following surgery and ongoing manual bladder expression was required at the time of manuscript preparation.

MMC and NTDs have been associated with other congenital abnormalities and genetic conditions (5, 32–34). There are multiple reports of dogs born with NTDs and other congenital malformations such as anencephaly, gastroschisis, and palatoschisis (32–34). In humans, Waardenburg syndrome, genitourinary and gastrointestinal disorders have been found to be associated with NTDs (5). There were no additional congenital abnormalities identified in the current case. When a congenital abnormality is identified, it is prudent to screen for others to help guide treatment and provide prognostic information (35).

Management of neural tube defects can be difficult with many pets having persistent deficits and incontinence despite medical or surgical intervention (1, 16–22). Prognosis with surgical intervention is difficult to predict due to the paucity of cases, but is likely associated with pre-operative neurologic status. At the time of submission, the cat described in this report had improved comfort, static paraparesis, and static incontinence following surgical intervention based upon repeat examination. It is important for veterinarians to recognize the clinical signs of NTDs in order to make responsible breeding recommendations and to improve case management.

## Data Availability

The raw data supporting the conclusions of this article will be made available by the authors, without undue reservation.
